# Up-Regulation of Rhoa/Rho Kinase Pathway by Translationally Controlled Tumor Protein in Vascular Smooth Muscle Cells

**DOI:** 10.3390/ijms150610365

**Published:** 2014-06-10

**Authors:** Jeehye Maeng, Vadim Sheverdin, Hyekyoung Shin, Insu Ha, Sun Sik Bae, Hsin-Fang Yang-Yen, Kyunglim Lee

**Affiliations:** 1 Graduate School of Pharmaceutical Sciences, College of Pharmacy, Ewha Womans University, Seoul 120-750, Korea; E-Mails: mengjeehye@hanmail.net (J.M.); vadimsheverdin@mail.ru (V.S.); hk1028@hanmail.net (H.S.); insu0623@gmail.com (I.H.); 2 Department of Pharmacology, Pusan National University—School of Medicine, Yangsan 626-870, Korea; E-Mail: sunsik@pusan.ac.kr; 3 Institute of Molecular Biology, Academia Sinica, Taipei 11529, Taiwan; E-Mail: imbyy@gate.sinica.edu.tw

**Keywords:** contraction, hypertension, myosin light chain (MLC), RhoA, translationally controlled tumor protein (TCTP), vascular smooth muscle cell (VSMC)

## Abstract

Translationally controlled tumor protein (TCTP), a repressor for Na,K-ATPase has been implicated in the development of systemic hypertension, as proved by TCTP-over-expressing transgenic (TCTP-TG) mice. Aorta of TCTP-TG exhibited hypercontractile response compared to that of non-transgenic mice (NTG) suggesting dys-regulation of signaling pathways involved in the vascular contractility by TCTP. Because dys-regulation of RhoA/Rho kinase pathway is implicated in increased vascular contractility, we examined whether TCTP induces alterations in RhoA pathway in vascular smooth muscle cells (VSMCs). We found that TCTP over-expression by adenovirus infection up-regulated RhoA pathway including the expression of RhoA, and its downstream signalings, phosphorylation of myosin phosphatase target protein (MYPT-1), and myosin light chain (MLC). Conversely, lentiviral silencing of TCTP reduced the RhoA expression and Rho kinase signalings. Using immunohistochemical and Western blotting studies on aortas from TCTP-TG confirmed the elevated expression of RhoA and increase in p-MLC (phosphorylated MLC). In contrast, down-regulation of RhoA and p-MLC were found in aortas from heterozygous mice with deleted allele of TCTP (TCTP^+/−^). We conclude that up-regulation of TCTP induces RhoA-mediated pathway, and that TCTP-induced RhoA plays a role in the regulation in vasculature. Modulation of TCTP may offer a therapeutic target for hypertension and in vascular contractility dysfunction.

## 1. Introduction

Translationally controlled tumor protein (TCTP), also called histamine releasing factor (HRF) and fortilin, is a protein highly conserved among many eukaryotic organisms with a high degree of homology in their primary sequence as well as tertiary structure (reviewed in [1]). TCTP’s multiple functions involve histamine-releasing activity (HRA) [2], regulation of cell cycle [3], apoptosis [4], and protein synthesis [5] and in pathologic conditions, such as tumorigenesis [6], allergy [2,7], and hypertension [8].

TCTP interacts with and suppresses the pump activity [9] of Na,K-ATPase, a fundamental transmembrane pump that takes part in the regulation of ionic homeostasis [10], as well as in signaling functions [11]. From the view of TCTP as a repressor for Na,K-ATPase, the pathophysiological role played by TCTP in the development of hypertension, as was the case with the cardiac glycoside ouabain [12], emerged with TCTP-over-expressing transgenic mice (TCTP-TG) [8]. TCTP-TG displayed essential hypertension at about 6 weeks after birth [8]. Reduced activity of sodium pump by TCTP induces the accumulation of sodium in cells which in turn increase the intracellular Ca^2+^ storage [8]. In addition, TCTP over-expression was found to increase the severity of atherosclerotic plaque formation, in a study with TCTP-TG mice crossed with apolipoprotein E knockout mice (ApoE KO), fed high fat diet [13], possibly through the up-regulation of proatherogenic factors including hemodynamic perturbation and inflammatory mediation [14,15].

Contraction of smooth muscle is governed by intracellular Ca^2+^ concentration that activates myosin light chain kinase (MLCK) and by the Ca^2+^ sensitivity of myofibrils mediated through the regulation of myosin light chain phosphatase (MLCP), which is partly controlled via Rho-associated kinase [16]. MLCP comprises subunits including protein phosphatase type 1 (a catalytic domain), myosin phosphatase target protein (MYPT-1, a myosin binding subunit), and a non-catalytic subunit with unknown function [17]. Loss of phosphate in MLC by MLCP induces smooth muscle relaxation whereas phosphorylation on MLC by MLCK induces vasocontraction [18]. Regulation of smooth muscle contraction therefore, depends on the phosphorylation the 20 kDa regulatory myosin light chain (MLC20) [19], whose activity relies on the relative actions of MLCK and MLCP.

Therefore up-regulation of RhoA/Rho kinase axis that induces the inhibition of MLCP appears to have a role in the regulation of vascular contractility in hypertension [20]. RhoA, a small GTPase, mediates Rho kinase activation, which in turn results in the phosphorylation of MYPT-1 of MLCP, rendering it inactive, thereby preserving the phosphorylated myosin light chain (p-MLC) and maintaining muscle contractions [20]. Mounting evidence suggests a role for RhoA/Rho kinase signaling in the increased muscle contractility [21] in the development of hypertension in humans [22]. This study aims to examine whether TCTP plays a role in the regulation of RhoA/Rho kinase pathway *in vitro* and *in vivo*.

## 2. Results

### 2.1. Translationally Controlled Tumor Protein (TCTP) Regulates the RhoA/Rho Kinase Pathway in Vascular Smooth Muscle Cells (VSMCs)

Over-expression of TCTP has been shown to induce systemic hypertension, in TCTP-TG mice and the aorta isolated from TCTP-TG displayed an increased contractility [8]. Since RhoA kinase is involved in Ca^2+^ sensitization of smooth muscle contraction [23], and RhoA-mediated pathway is implicated in the pathophysiology of hypertension [16], we examined the possible role of RhoA signaling in TCTP-induced VSMC contraction, especially whether and how the components of RhoA/Rho kinase signaling pathway involved in the Ca^2+^ sensitization, *in vitro*. Rho kinase activity was measured as increase in phosphorylated myosin phosphatase subunit (p-MYPT1).

**Figure 1:**
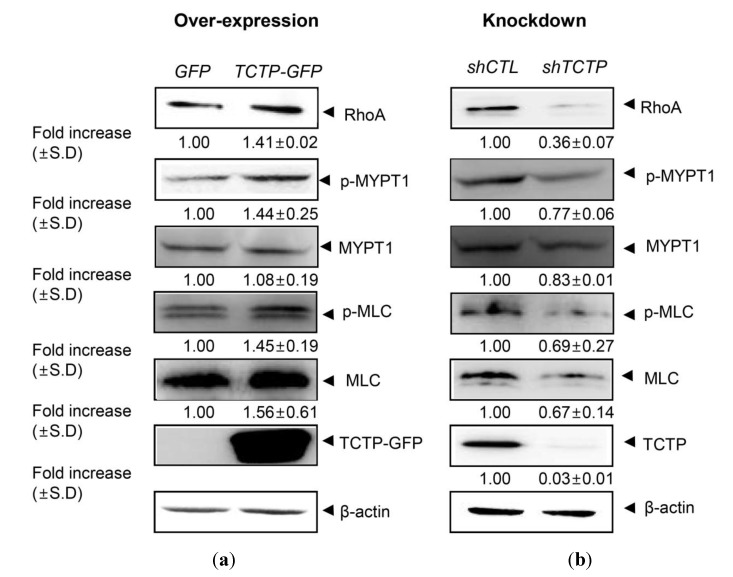
Role of translationally controlled tumor protein (TCTP) in the RhoA-mediated signaling pathway in vascular smooth muscle cells (VSMCs). (**a**) Over-expression of TCTP in primary cultured VSMCs were studied using adenoviral infection at a 100 MOI of adGFP and adTCTP-GFP. VSCMs were serum-starved before experiment for 24 h and lysed cells were used. Following sodium dodecyl sulfate-polyacrylamide gel electrophoresis (SDS-PAGE), proteins were detected using antibodies including anti-RhoA, -p-MYPT1, -MYPT1, -p-MLC, -MLC, -GFP, and -β-actin-specific antibodies; (**b**) Lentiviral silencing of TCTP expression in VSMCs was achieved, as described in Material and Methods. After serum starvation for 24 h, cell lysates were prepared. Protein contents in cell lysates were detected by Western blotting using indicated antibodies. Band intensities were measured using Image J software (National Institute of Health, Bethesda, MD, USA) and normalized to β-actin. Fold increase were expressed as mean ± SD (*N* = 2).

As shown in Figure 1A, TCTP over-expression resulted in increases in phosphorylated myosin phosphatase subunit (p-MYPT1), a subtle elevation in myosin light chain (MLC) expression, and an up-regulation of phosphorylation of MLC (p-MLC) reflecting over-expression of RhoA. Conversely, silencing of TCTP in VSMCs decreases in RhoA, phospho-MYPT1, p-MLC, and MLC signals (Figure 1B), suggesting a correlation between the expression of TCTP and the regulation of RhoA/Rho kinase-mediated signaling pathways *in vitro* that are involved in the regulation of contractile response. This in turn implicates up-regulation of RhoA expression in the hypercontractile response of aorta of TCTP-TG mice.

### 2.2. Up-Regulation of RhoA Expression and Phosphorylated Myosin Light Chain (p-MLC) in TCTP-Over-Expressing Transgenic (TCTP-TG) Mice

**Figure 2:**
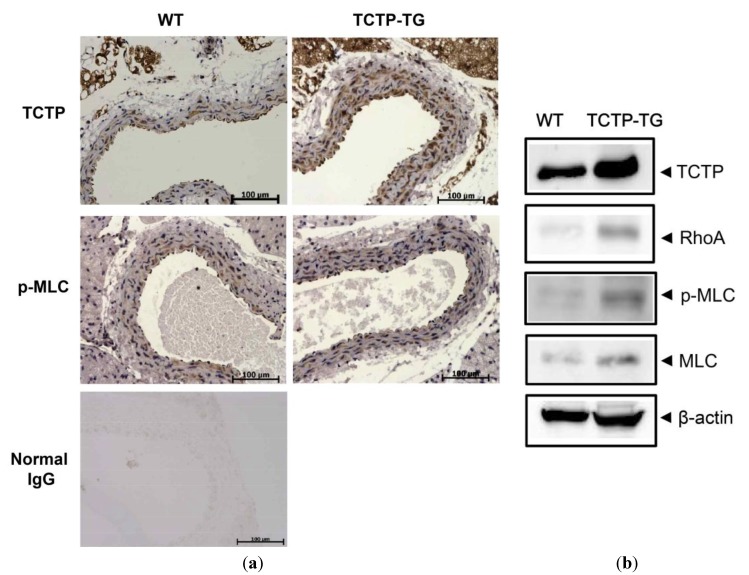
Role of TCTP in the RhoA-mediated signaling pathway in TCTP-transgenic (TCTP-TG) mice. (**a**) Representative photomicrographs of immunohistochemistry (IHC) detecting TCTP and p-MLC in the aorta from wild-type (WT) and TCTP-TG mice. Immunostaining of aortas from WT and TCTP-TG were performed with antibodies detecting TCTP and phosphorylated MLC (p-MLC). Nuclei were counterstained blue with hematoxylin reagent (*N* = 3); (**b**) Western blot analysis of aortic tissues was done as described in Materials and Methods using anti-TCTP, -RhoA, -p-MLC, -MLC, and -β-actin-specific antibodies.

To confirm the possible alteration of RhoA pathway by TCTP *in vivo*, we investigated RhoA expression and phosphorylation of MLC in aorta from TCTP-TG mice using immunohistochemistry (IHC) and Western blotting. This study showed increased expression of TCTP and phosphorylation of MLC in aortas from TCTP-TG, when compared to their control non-transgenic mice (NTG) (Figure 2A). There was negligible immunoreactivity when the samples were probed with normal IgG (herein used as a negative control). TCTP was detected in all the layers of entire vasculature, while p-MLC was mainly localized in VSMCs that comprise the medial layer.

Western blotting using relevant antibodies performed on identical aortic tissues confirmed elevated expression of TCTP and p-MLC signals in TCTP-TG mice shown in Figure 2A. Expression of TCTP and p-MLC were elevated in aorta derived from TCTP-TG mice compared to NTG. RhoA and MLC expression also increased in TCTP-TG, confirming the TCTP-induced RhoA signaling activation in the vasculature (Figure 2B).

### 2.3. Down-Regulation of RhoA Expression and p-MLC in TCTP^+/−^ Mice

Aortic tissues from TCTP^+/−^ mice were used for verifying the influence of TCTP down-regulation *in vivo*, and were also investigated by IHC. Knock-out mice devoid of *TCTP* genes (*TCTP*^−/−^) are embryonically lethal while heterozygous mice that have deletions of one allele of *TCTP* (*TCTP*^+/−^) are viable [24]. It is reported that TCTP^+/−^ mice are fertile and are similar to their WT littermates in morphology [24].

**Figure 3:**
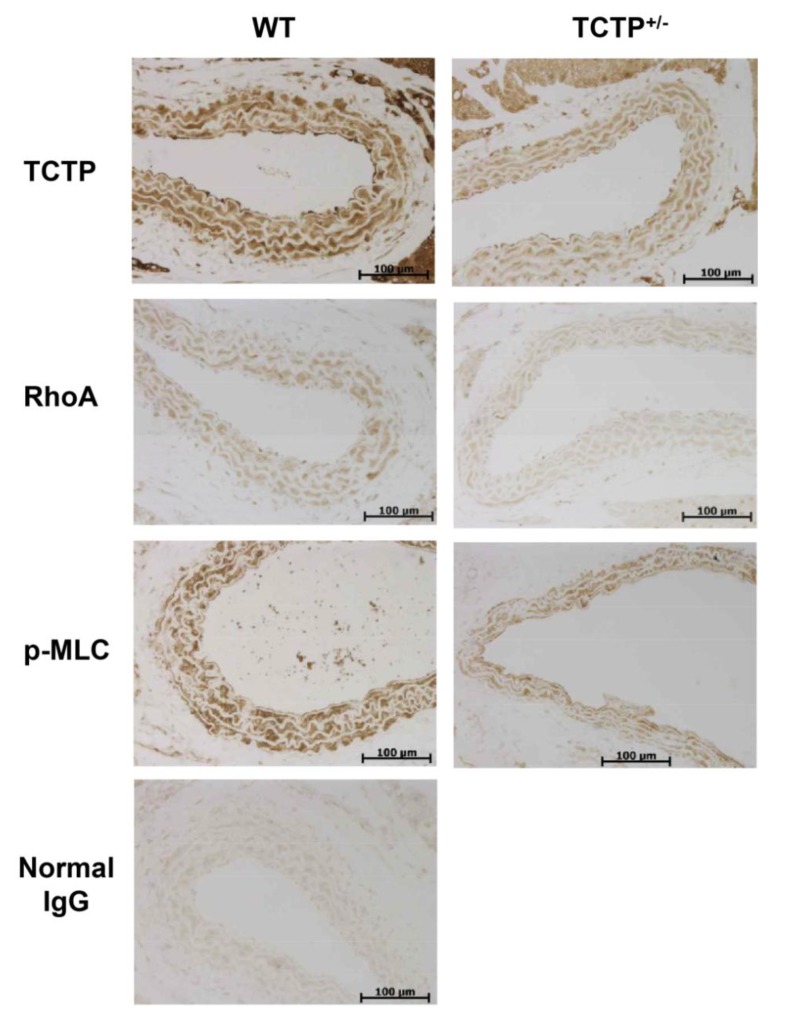
Role of TCTP in the RhoA-mediated signaling pathway in TCTP^+/−^ mice. Representative photomicrographs indicating immunoreactivity of TCTP, RhoA, and p-MLC in the sections of aortic tissues from WT and TCTP^+/−^ mice. IHC was performed using anti-TCTP, -RhoA, and p-MLC antibodies (*N* = 4–5).

Aorta from TCTP^+/−^ mice displayed immunostained TCTP in intima, media, and adventitia layers (Figure 3), the entire layer of media, except for internal elastic lamina, reacting with anti-TCTP, -RhoA, and -p-MLC antibodies. The immunolocalization of RhoA in IHC through all layers of aorta, is consistent with previous report [25], was reduced in the aortas form TCTP^+/−^ mice that exhibit reduced TCTP expression (Figure 3). Furthermore, it was found that TCTP^+/−^ mice exhibit reduced phosphorylation of MLC *in vivo* (Figure 3). These findings suggest that down-regulation of TCTP is involved in the reduction in the RhoA and p-MLC *in vitro* and *in vivo*. Taken together, TCTP seems to regulate RhoA/Rho kinase signaling pathways, implying that RhoA, pivotal player in the vasocontraction, is involved in the pathogenesis of TCTP-induced hypertension in TCTP-TG mice.

## 3. Discussion

Abnormal contractility of smooth muscle, a hallmark for hypertension, is also found in mice that over-express TCTP [8]. In general, contraction of VSMC is mainly governed by cytosolic Ca^2+^ that induces MLC phosphorylation through the MLCK action. In addition, Ca^2+^ sensitivity of myofilaments, which is regulated through the inhibition of MLCP by RhoA/Rho kinase pathway, also takes part in the vascular contractility [16,18,19,26]. In this pathway, small GTPase RhoA and its downstream target, Rho kinase (ROCK) regulate MLCP, contributing to Rho/ROCK-mediated Ca^2+^ sensitization [16]. Therefore, up-regulation of RhoA family of proteins and its downstream pathway, play important roles in the pathogenesis of hypertension [26]. These are also found in the aorta of hypertensive animal models, such as spontaneously hypertensive rat (SHR), and deoxycorticosterone acetate (DOCA) salt-induced hypertensive rat [26]. Conversely, Y-27632, a Rho kinase inhibitor, considerably reduces blood pressure in hypertensive rats, such as SHR [16]. These observations suggest the possible usefulness of inhibitory strategies for RhoA/Rho kinase pathway, in the therapy of hypertension. Some studies using the first generation ROCK inhibitor, fasudil, indicated the usefulness of Rho kinase inhibitors in the hypertension including pulmonary arterial hypertension [27,28].

In our previous study, the dose-response curve of KCl-induced contraction of aorta showed pronounced contraction at a low concentration of K^+^ in aorta from TCTP-TG, compared to that of wild-type mice [8]. Since KCl has been shown to induce smooth muscle contraction not only via activation of voltage-operated Ca^2+^ channels (VOCC) but also by Ca^2+^ sensitization involving RhoA kinase [29], increased contractile responses in vascular smooth muscles from TCTP-over-expressing transgenic mice [8], can result from the abnormal regulation of RhoA pathway by TCTP over-expression.

In this study, TCTP appears to mediate the expression of RhoA, a crucial component of vasocontraction regulation, thereby inducing Ca^2+^ sensitization in primary cultured vascular smooth muscle cells. Phosphorylation at Ser-19 of myosin light chain 20 (MLC20, also named MRLC), in SMC permits the cross-bridging of myosin(II)-actin. Therefore, elevated expression of RhoA and phosphorylation at Ser-19 of MLC20/p-MLC in TCTP-over-expressing VSMCs (Figure 1A) and aorta of TCTP-TG (Figure 2) suggests the possible involvement of RhoA-mediated signaling in TCTP-induced vasocontraction. A recent report that RhoA is up-regulated in ovarian cancer cells which express increased levels of TCTP [30], supports a possible role for RhoA-TCTP interaction in biological processes. How TCTP activates the RhoA pathway and MLC phosphorylation, including its potential effects on the MLCK, need to be determined.

In addition, it would be interesting to examine whether TCTP-induced regulation of RhoA/Rho-kinase pathway might be responsible for the blood pressure elevation in TCTP-TG. To this end, the effect on BP in TCTP-TG mice of Rock inhibitor treatment will be studied and confirmed in our future research. In this context, agents reducing the abnormally elevated intracellular TCTP in VSMCs and thereby down-regulate RhoA signaling might help in the therapy of systemic hypertension. Studies on the effects of active forms of RhoA, RhoA-GTP, employing RhoA activation assay, and on the expression of its downstream effector, Rho-kinase, are underway in our laboratory in efforts to dissect the activation of RhoA/Rho-kinase pathway by TCTP. Further investigation on TCTP-induced RhoA regulation under vasoconstricting stimuli such as NE, 5-HT, and high K^+^, will be of interest.

## 4. Experimental Section

### 4.1. Animal Studies

TCTP-over-expressing transgenic mice were generated using the pCAGGS-TCTP cDNA constructs, as described previously [8]. Hemizygous mice that have only a single copy of *TCTP* gene, *TCTP*^+/−^, were kindly provided by Hsin-Fang Yang-Yen (Institute of Molecular Biology, Academia Sinica, Taipei, Taiwan) [24]. All animal studies were performed in compliance with the Guide for the Care and Use of Laboratory Animals of National Institutes of Health and were approved by the Ewha Institutional Animal Care and Use Committee (IACUC) of Ewha Womans University (Permit number 2013-01-064, 1 July 2013).

### 4.2. Cell Culture

HEK293FT cells were from Invitrogen (Carlsbad, CA, USA). Vascular smooth muscle cells (VSMCs) were isolated from thoracic aorta of Sprague-Dawley (SD) rats as described below. All cells were maintained in Dulbecco’s modified Eagle’s medium (DMEM, Gibco BRL, Grand Island, NY, USA), supplemented with 10% fetal bovine serum (FBS, Gibco), and penicillin/streptomycin (Gibco) at 37 °C under humidification with 5% CO_2_.

### 4.3. Isolation of Vascular Smooth Muscle Cell (VSMC) from Rat Aorta

Tissue explanting method with slight modification was used to isolate the rat aortic smooth muscle cells from the thoracic aorta of 3–5 weeks old SD rats. Briefly, thoracic aorta of SD rat was aseptically isolated under anesthesia, fat and connective tissues surrounding aorta were trimmed, and the aortic vessels were longitudinally cut into 3–5 mm lengths. The cut pieces were explanted with lumen sides down on culture dishes and were incubated with DMEM media containing 10% FBS. Culture medium was freshly changed every other day. Following the 6 days of incubation, tissues were carefully removed from the culture dishes. Sprouted VSMCs at the bottom of the dishes were harvested for further experiments. Their SMC lineage was confirmed by the Western blotting using antibodies detecting α-smooth muscle actin (α-SMA, Sigma-Aldrich, St Louis, MO, USA) and calponin (Sigma).

### 4.4. Virus Induction of TCTP Over-Expression

To over-express the *TCTP* gene in VSMCs, VSMCs were infected with adenovirus containing with AdGFP- and adTCTP-GFP-constructs at a multiplicity of infection (MOI) of 100. Following incubation of adenovirus infected VSMC in serum-free DMEM, for 3 h, the cells were incubated with serum-containing DMEM for 48 h. TCTP expression in VSMCs was inhibited with lentiviral-mediated delivery of shTCTP. Toward this end, pLKO-shTCTP was constructed using *Age*I and *Eco*RI restriction enzyme sites of pLKO vector, with primers (forward: 5'-CCGGTGAAGGTACCGAAAGCACAGTACTCGAGTACTGTGCTTTCGGTACCTTCTTTTTG-3'; reverse: 5'-AATTCAAAAAGAAGGTACCGAAAGCACAGTACTCGAGTACTGTGCTTTCGGTACCTTCA-3'). HEK-293T cells were transfected with pLKO or pLKO-shTCTP vectors with pVSV-G and Δ8.9 using calcium phosphate method. Cell culture supernatants containing viruses were harvested for infection. The virus-containing media was supplemented with Polybrene (Sigma), and then filtered through a 0.45 μm syringe-driven to remove debris and the filtered media was added to the culture of VSMCs. After incubation with virus particles, infected cells were selected through treatment of puromycin (Sigma). The infected cells were serum-starved for 24 h and used for further analysis.

### 4.5. Western Blotting

After washing the cells with phosphate-buffered saline (PBS), cells were collected by scraping from the cell culture vessel. Cells were incubated with lysis buffer (20 mM Tris, pH 7.5, 135 mM NaCl, protease inhibitor, phosphatase inhibitor cocktail (Sigma), 1% Triton X-100) on ice and sonicated for a short time and centrifuged at 12,000 rpm for 10 min at 4 °C. Protein concentration of the cell lysate was analyzed by Bradford assay (Bio-Rad Laboratories, Hercules, CA, USA). Samples containing cellular proteins were constituted in SDS sample buffer and boiled. Samples were resolved by sodium dodecyl sulfate-polyacrylamide gel electrophoresis (SDS-PAGE). After transferring the proteins from the gel to nitrocellulose (NC, Whatman, GE Healthcare Life Sciences, Piscataway, NJ, USA) or to polyvinylidene difluoride membrane (PVDF, Amersham Pharmacia, Piscataway, NJ, USA), the membranes were blocked and probed with primary antibodies, including anti-α-smooth muscle actin (α-SMA, Sigma), -calponin (Sigma), -RhoA (SantaCruz Biotechnology Inc., Santa Cruz, CA, USA or Bioworld Technology Inc., St. Louis Park, MN, USA), -MYPT-1 (Covance, Berkeley, CA, USA), -phospho-myosin phosphatase target subunit 1 (p-MYPT-1, Millipore, Billerica, MA, USA), -myosin light chain (MLC, Cell Signaling Technology Inc., Beverly, MA, USA), -phospho-myosin light chain (p-MLC, Cell Signaling Technology Inc.), -GFP (SantaCruz Biotechnology Inc.), -β-actin (Cell Signaling Technology Inc.), and -TCTP (Lab Frontier, Seoul, Korea). This was followed by incubation with a horseradish peroxidase (HRP)-conjugated secondary antibody (Bio-Rad). Protein on membrane was detected using an enhanced chemiluminescence (ECL, Amersham Pharmacia) reagent under the LAS-3000 UV Products Imaging system (Fujifilm, Tokyo, Japan).

### 4.6. Tissue Western Blotting

The aortic tissues were homogenized in ice-cold buffer containing protease inhibitor cocktail tablet (Roche, Indianapolis, IN, USA), 50 mM Tris–HCl (pH 7.4), 150 mM NaCl, 1 mM EDTA, 2 mM Na_3_VO_4_, 1 mM NaF, 0.25% deoxycholate, and 1% Triton X-100 and centrifuged at 15,000× *g* for 10 min at 4 °C. Protein contents of the supernatants were determined by BCA protein assay (Pierce, Rockford, IL, USA) as follows. Ten micro grams of samples were diluted with sample buffer (100 mM Tris–HCl, 2% SDS, 1% β-mercaptoethanol, 2% glycerol, 0.01% bromophenol blue), followed by incubation at 95 °C for 10 min. The protein extract was separated by SDS-PAGE and proteins were transferred onto NC membranes (Whatman). Washed membranes were blocked in a blocking solution for 1 h at room temperature, and incubated with primary antibodies at 4 °C for overnight and washed. The membranes were then incubated with HRP-conjugated secondary antibody. The protein bands were developed by enhanced chemiluminescence (ECL, Amersham) and LAS-3000 systems (Fujifilm).

### 4.7. Immunohistochemistry

After dissection, the aortic tissues were excised and immersed in phosphate buffer supplemented with 10% formalin. Samples were fixed for 24 h, dehydrated in graded ethanol embedded in paraffin blocks and dissected into 5–6 µm sections. The tissue sections were then placed on glass slides deparaffinized in xylene followed by rehydration. They were then boiled with sodium citrate buffer (pH 6.0) for 20 min, to retrieve the epitopes. The endogenous peroxidase in the samples was blocked with 1% hydrogen peroxide solution. Immunoreactivity in samples was visualized using ImmPRESS Reagent (Vector, Burlingame, CA, USA) following the manufacturer’s instructions. The following dilutions of the primary antibodies were used: anti-RhoA rabbit polyclonal antibody (Bioworld Technology Inc., St. Louis Park, MN, USA, BS1782, 1:50), anti-p-Myosin Light Chain 2 rabbit polyclonal antibody (Cell Signaling, #3671, 1:200), anti-TCTP rabbit polyclonal antibody (Abcam, Cambridge, MA, USA, ab37506, 1:1000). Samples for negative control were probed with normal rabbit IgG (Abcam, Ab27478). For visualization, 3,3'-diaminobenzidine (DAB, Sigma) was used. Harris Hematoxylin (Sigma) was treated for nuclear counter stain. All sections were mounted with cover slip using Permount (Fisher Scientific, Fair Lawn, NJ, USA), and then observed and photographed under the electron microscope.

### 4.8. Assay of Rho Kinase Activity in VSMC

The smooth muscle cell lineage of primary cultured VSMC was confirmed by immunoblotting with α-SMA and calponin (data not shown). To manipulate TCTP expression in VSMC, viral-mediated gene delivery systems were applied using retroviral infection for over-expression and lentiviral gene silencing for knock-down of *TCTP*. Following serum starvation of primary cultured VSMCs, TCTP-over-expressed and -down-regulated cells were analyzed for their potential induction for expression and activation of players involved in RhoA/Rho kinase pathway using Western blotting. Rho kinase activity was measured as increase in phosphorylated myosin phosphatase subunit (p-MYPT1) using anti-p-MYPT-antibody.

### 4.9. Statistical Analyses

Band intensities obtained by Western blotting were quantitated using Image J software (National Institute of Health, Bethesda, MD, USA). After normalizing band intensities with that of β-actin, fold increases are expressed as mean ± standard deviation (SD).

## 5. Conclusions

This study suggests that TCTP plays a role in the regulation of vascular contractility, via up-regulation of RhoA/Rho kinase pathway. TCTP over-expression-induced RhoA activation indicates that silencing of cellular TCTP may offer therapeutic approaches for the regulation of hypertension by inhibiting RhoA/Rho kinase pathway and modulating vascular contractility. Clearly, further studies are needed to confirm the role of TCTP-mediated RhoA regulation in the development of hypertension.
